# Co-application of high temperature biochar with 3,4-dimethylpyrazole-phosphate treated ammonium sulphate improves nitrogen use efficiency in maize

**DOI:** 10.1038/s41598-021-85308-0

**Published:** 2021-03-11

**Authors:** Niguss Solomon Hailegnaw, Filip Mercl, Martin Kulhánek, Jiřina Száková, Pavel Tlustoš

**Affiliations:** Department of Agro-Environmental Chemistry and Plant Nutrition, Faculty of Agrobiology, Food and Natural Resources, Czech University of Life Sciences Prague, Kamýcká 129Prague 6, 16500 Suchdol, Czech Republic

**Keywords:** Plant sciences, Environmental sciences

## Abstract

This study aimed on the increasing nitrogen use efficiency (NUE) of maize via the use of high temperature produced biochar (700 °C). Maize was grown to maturity on two contrasting soils (acidic Cambisol and neutral Chernozem) in pots with a treatment of biochar co-applied with ammonium sulphate stabilised by a nitrification inhibitor (3,4-dimethylpyrazole-phosphate, DMPP) or un-stabilised. The combination of biochar with ammonium sulphate containing DMPP increased maize biomass yield up to 14%, N uptake up to 34% and NUE up to 13.7% compared to the sole application of ammonium sulphate containing DMPP. However, the combination of biochar with un-stabilised ammonium sulphate (without DMPP) had a soil-specific influence and increased maize biomass only by 3.8%, N uptake by 27% and NUE by 11% only in acidic Cambisol. Further, the biochar was able to increase the uptake of phosphorus (P) and potassium (K) in both stabilised and un-stabilised treatments of ammonium sulphate. Generally, this study demonstrated a superior effect from the combined application of biochar with ammonium sulphate containing DMPP, which improved NUE, uptake of P, K and increased maize biomass yield. Such a combination may lead to higher efficiency of fertilisation practices and reduce the amount of N fertiliser to be applied.

## Introduction

Nitrogen (N) is usually the most growth-limiting nutrient of crops, so crop production is highly dependent on the N soil supply capacity^1^. From N sources, plants can take up N mainly in the form of ammonium (N–NH_4_^+^) and nitrate (N–NO_3_^−^). However, their availability in soils is limited and accounts for only 2% of the total soil N content. Due to the growing demand in crop production, the use of inorganic N fertilisers has dramatically increased over the past 50 years^2^, resulting in crop yields enhanced by 30–50%^3^. However, overall nitrogen use efficiency (NUE) of applied fertilisers by cereals is typically ranging from 30 to 50%^4^.

Such low NUE leads to environmental issues^5^ and fertilisers are applied in an excessive amount to cope with the low NUE. This excessive use of inorganic fertilisers causes numerous problems related to soil chemistry, root growth, losses of nitrogen and may result in soil degradation. Low NUE is mainly caused by the decline in the availability of nitrate and ammonium at the later stages of crop growth due to the losses of these species through volatilisation, denitrification and leaching^6^. Recently, N fertilisers containing nitrification inhibitors are in use to reduce the fast oxidation of NH_4_^+^ and its subsequent leaching to reduce the N losses from soil^7–9^. Biochar (BC) is among the materials often cited as the effective soil additive being able to induce N sorption and reduce losses^10–12^. Moreover, BC application has been recommended for the restoration of degraded and acidified soils due to the excessive use of N fertilisers^13^. BC is also known to increase soil pH^14^, increase the soil content of base cations (Ca^2+^, Mg^2+^ and K^+^) and increase the cation exchange capacity (CEC) of soils^15–18^. The reduction of both NH_3_ volatilisation and NO_3_^−^ leaching due to the adsorption effect of BC has also been reported^18–21^. This effect of BC arises from the alkaline components of BC, including ash and carbonates of Ca^2+^, Mg^2+^ and K^+^^22,23^, unique physical properties (high porosity and surface area) and chemical properties (negatively and positively charged surface)^24^. Ammonium sulphate can be more efficiently combined with biochar than other N forms of fertilizers to improve NUE. Urea-N is available to plants after the hydrolysis, increasing soil pH^25^, so its co-application with biochar could further increase soil pH^15,26–28^ making nutrients like phosphorous less available. Fertilizers containing both ammonia and nitrate forms are less effective to regulate N soil transformation^25^.

Hence, we hypothesised that soil application of biochar in the combination with ammonium sulphate (AS) stabilised with 3,4-dimethylpyrazole-phosphate (DMPP) could increase the nitrogen use efficiency (NUE) and yield of maize biomass. Further, this study aimed to elucidate the mechanisms and chemical changes caused by the co-application of these materials.

## Material and methods

### Soil and biochar sources and characteristics

Two soils with desired properties were selected based on our previously published work^20,29^, where an identical biochar (wood chips pyrolysed at 700 °C) was used. The relatively higher biochar production temperature (700 °C) was preferred due to the need for producing relatively stable biochar with relatively lower ammonium and a higher nitrate sorption capacity. Out of the ten soils used in the previous studies, two contrasting soils were chosen: (1) Chernozem (silt clay loam; locality Suchdol, Czech Republic) a soil characterised by a neutral pH and a decline in the concentration of exchangeable Ca and cation exchange capacity (CEC) after the application of BC and (2) Cambisol (silt loam; locality Žamberk, Czech Republic) soil was selected for its acidic pH and an increase in the concentration of exchangeable Ca and CEC after BC application. Detailed characteristics of the soils and BC are presented in Table 1.

**Table 1:** Selected physiochemical properties of soils and biochar (Hailegnaw et al., 2019b).

Properties	Suchdol	Žamberk	Biochar
Localization	50°07′40″N, 14°22′35″E	50°08′40″N, 16°30′50″E	–
Soil type	Chernozem	Cambisol	–
pH^§^	6.90	4.80	9.50
CEC (mmol kg^−1^)	249.3 ± 4.0	74.9 ± 3.7	102 ± 5.2
Total N (%)	0.16 ± 0.00	0.20 ± 0.00	0.40 ± 0.02
Organic carbon (%)	1.61 ± 0.1	1.60 ± 0.0	–
C/N ratio	13.2 ± 0.16	9.96 ± 0.16	219.98 ± 12.9
DOC (mg kg^−1^)^§^	13.4 ± 4.3	63.6 ± 2.0	–
N–NH_4_^+^ (mg kg^−1^)^§^	5.7 ± 0.8	23.5 ± 3.2	–
Available P (mg kg^−1^)^§^	6.23 ± 0.17	2.36 ± 0.07	n.d
Available K (mg kg^−1^)^§^	65 ± 0.21	31.7 ± 0.59	2278 ± 66
Available Mg (mg kg^−1^)^§^	77 ± 0.86	21.1 ± 0.2	192 ± 11
Available S (mg kg^−1^)^§^	25.4 ± 1.81	17.2 ± 0.1	32 ± 1.6
P (mg kg^−1^)^¥^	955 ± 12.5	530 ± 12.0	496 ± 0.22
K (mg kg^−1^)^¥^	6680 ± 113	3816 ± 158	2670 ± 225
Ca (mg kg^−1^)^¥^	9987 ± 64.8	1607 ± 32.8	6676 ± 586
Mg (mg kg^−1^)^¥^	4940 ± 12.9	2332 ± 68.0	1176 ± 71.9
S (mg kg^−1^)^¥^	227 ± 13.9	150 ± 5.4	127 ± 11.6
Exch.Ca^2+^ (mmol kg^−1^)	253 ± 3.7	72 ± 0.6	176 ± 13.5
Exch. K^+^ (mmol kg^−1^)	4.6 ± 0.1	1.6 ± 0.1	50.4 ± 0.3
Exch. Mg^2+^ (mmol kg^−1^)	11.8 ± 0.3	2.7 ± 0.1	23.2 ± 7.3
Sand (%)	13.16	26.08	–
Silt (%)	60.05	59.82	–
Clay (%)	26.77	14.08	–
Textural class	Silt clay Loam	Silt Loam	–

^§^0.01 mol L^−1^ CaCl_2_ extract, n.d: not detected (0.05 mg kg^−1^), Exch.: exchangeable.

^¥^Pseudo-total content.

### Pot experiment

The pot experiment was set up using 5 kg (dry weight) soil in 6-L pots in a precipitation-controlled vegetation hall. Nine treatments were set up (Table 2) to achieve the aim of the study in a completely randomised design for each soil. Each treatment was prepared in four replicates.

**Table 2:** The experimental design set up.

No ammonium sulphate (NoAS)	Un-stabilized ammonium sulphate (USAS)	Stabilized ammonium sulphate with DMPP (SAS)
Control (no biochar)	No biochar + 1.0345 g N from USAS	No biochar + 1.0345 g N from SAS
1% biochar	1% biochar + 1.0345 g N from USAS	1% biochar + 1.0345 g N from SAS
2% biochar	2% biochar + 1.0345 g N from USAS	2% biochar + 1.0345 g N from SAS

The nitrogen fertilisation rate represented 207 mg N kg^−1^ of soil and corresponded roughly with the N application rate of 600 kg N ha^−1^ in field conditions. In this study, stabilised ammonium sulphate (SAS) was bought from COMPO EXPERT GmbH (Germany) with the product trade name NovaTec Solub 21 having (0.205% of 3,4-dimethylpyrazole-phosphate (DMPP) and 21% N). The corresponding un-stabilised ammonium sulphate (USAS) treatment was fertilised using ammonium sulphate ((NH_4_)_2_SO_4_; 21% N) from the AGRO CS Group (Řikov, Czech Republic). Fertilisers were applied in the form of powder and were thoroughly mixed with soil. After preparing all the treatments, five maize seeds were sown per pot and thinned to three plants per pot two weeks after sowing. Each pot was regularly irrigated to 60% of the soil maximum water holding capacity. Soil solution was collected over the vegetation period using Rhizon MOM suction cups as described by Refs.^30,31^. Maize aboveground biomass was harvested 115 days after sowing, oven-dried (65 °C) and ground to a fine powder before analyses. After the harvest, soil samples were collected and analysed for the available content of mineral N.

### Soil analyses

The measurements of soil and biochar pH were done using an Argus pH meter (Sentron, Netherland) with a transistor CupFET probe after the extraction of samples with 0.01 M CaCl_2_ (w/v = 1/5). The available content of nutrients in both soil and biochar were determined by the use of inductive coupled plasma-optical emission spectrometry (ICP-OES; Agilent 720, Agilent Technologies Inc., Santa Clara, CA) after the extraction of samples with 0.01 M CaCl_2_ in 1:10 (w/v) for 2 h^32^. The available content of inorganic N (nitrate and ammonium nitrogen) were measured by the Skalar San Plus System continuous flow segmented analyser (Skalar, Netherlands) after extraction of samples with 0.01 M CaCl_2_ (w/v = 1/10) for 2 h^33^. The total content of C and N were determined by the use of a CHNS elemental analyser (Vario MACRO cube system GmbH, Hanau, Germany). The total organic carbon (TOC) was determined according to Sims et al.^34^, i.e. spectrophotometrically following the oxidation of organic matter (OM) with K_2_Cr_2_O_7_. Determination of cation exchange capacity was done according to Gillman et al.^35^ by a three-step saturation of samples (1 h for each agitation) with 0.1 M BaCl_2_ solution and collecting the extracts for determination of exchangeable cations (Ca^2+^, K^+^ and Mg^2+^) by ICP-OES. The pellet remaining after extraction was used for subsequent release of Ba^2+^, where it was agitated with 0.02 M MgSO_4_ for two hours, and then CEC was calculated based on the amount of Mg^2+^ retained by the soil or biochar. The pseudo-total contents of elements (P, Ca, K, Mg and S) in both soils and biochar were determined by ICP–OES after microwave assisted *aqua regia* extraction^36^.

### Plant analysis

The concentration of nutrients in maize biomass was determined after the digestion of plant samples with concentrated HNO_3_ (65% v/v; Analytika) and H_2_O_2_ (30% v/v; Analytika) in an Ethos 1 microwave-assisted wet-digestion system (MLS, Leutkirch, Germany), and P, S, Mg and Ca concentrations in the digests were determined by ICP-OES. The concentrations of K were determined using flame atomic absorption spectrometry (F-AAS; Varian AA285FS, Varian, Australia). The total concentrations of N in maize tissue were determined by the Kjeldahl method (Vapodest 50 s, Gerhardt, Germany).

### Statistical analysis

All statistical analyses were performed using SPSS 17.0 software. The effect of biochar was determined by one-way analysis of variance (ANOVA) at *p* < 0.05 followed by the Tukey test to assess the effect of the individual treatments. The interactions of the variables (e.g. biochar application, fertiliser application and soil) on maize biomass and yield component were analysed by a multivariate analysis of variance, MANOVA. The repeated measure analysis of variance rANOVA was implemented to describe the within-subject effect of sampling time and between-subject effect of soil, biochar, fertiliser and their interaction on the pH and nutrient content of soil solution. The uptake of nutrients (mg per pot) by maize was calculated as Eq. (1).

1 $$ Nutrient\;uptake = Maize\;dry\;matter\;yield \times shoot\;nutrient\;concentration $$

where maize dry matter yield was in g per pot and shoot nutrient concentration was in mg g^−1^.

Nitrogen use efficiency (NUE) and sulphur use efficiency (SUE) was calculated according to Eq. (2).

2 $$ NUE{\mkern 1mu} \left( \% \right) = \left[ {\left( {N_{FT} - N_{CT} } \right)/N_{ap} } \right] \times 100 $$

where *N*_*FT*_ is the N or S uptake in fertilised treatment, *N*_*CT*_ is the N or S uptake in corresponding control, non-fertilised treatment, and *N*_*ap*_ is the amount of N or S applied in a pot in the form of ammonium sulphate.

## Results

### Biomass yield

**Figure 1:**
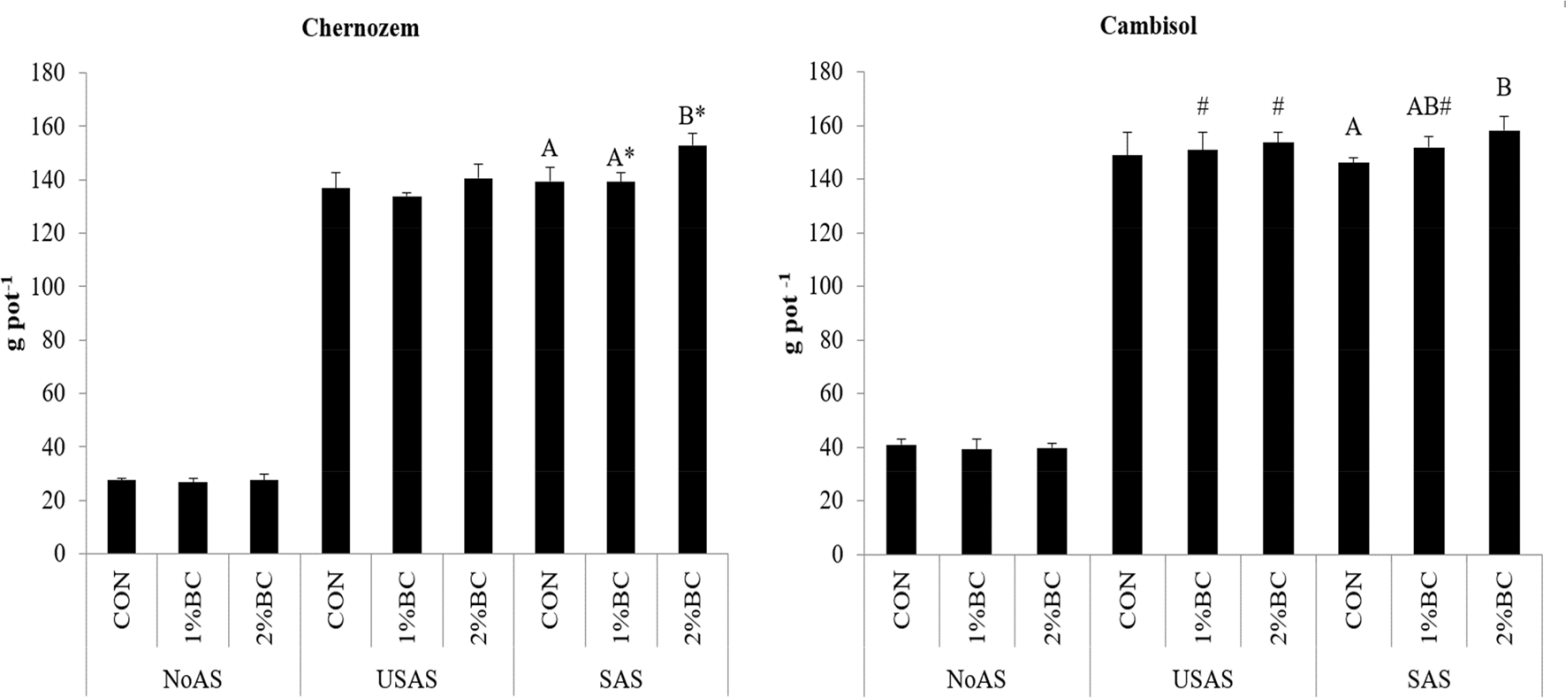
The yield of maize aboveground biomass at full maturity. USAS (un-stabilized ammonium sulphate) and SAS (stabilized ammonium sulphate with DMPP). Different upper-case letters indicate a significant difference between variants within the same treatments of the same soil. *Represents a significant difference along biochar treatments of SAS and USAS (pair wised t-test between 1% BC of USAS and 1% BC of SAS and between 2% BC of USAS and 2% BC of SAS). ^#^Represents significant difference of pair wise t-test along different soils of similar treatments (CON of USAS Chernozem with CON of USAS Cambsiol and likewise). *NoAS* No ammonium sulphate, *CON* control, *1% BC* 1% biochar, *2%BC* 2% Biochar.

The biomass yield in the SAS and USAS treatments was up to 5 times higher than in the NoAS treatments. The effect of biochar on maize biomass was not significant in the case of NoAS and USAS treatments (Fig. 1). However, in the SAS, the application of 2% biochar significantly increased maize biomass by 10 and 8% in the Chernozem and Cambisol soils, respectively. In the Chernozem with the 2% application, the biochar with SAS showed significantly higher biomass than USAS treatment (Fig. 1). Moreover, the biomass yield of maize was higher in the Cambisol in the 1% BC and 2% BC treatments of USAS and the 1% BC treatments of SAS than in the corresponding treatments of the Chernozem. The main factor influencing the maize biomass yield was the application of fertiliser (F = 5391, *p* > 0.001), followed by type of soil (F = 131, *p* > 0.001) and the application of biochar (F = 11.9, *p* > 0.001), see supporting information (SI S1). The interaction effect of biochar and fertiliser was also a source of significant (F = 5.39, *p* = 0.001) effect.

### The uptake of nutrients by maize

#### Nitrogen

**Figure 2:**
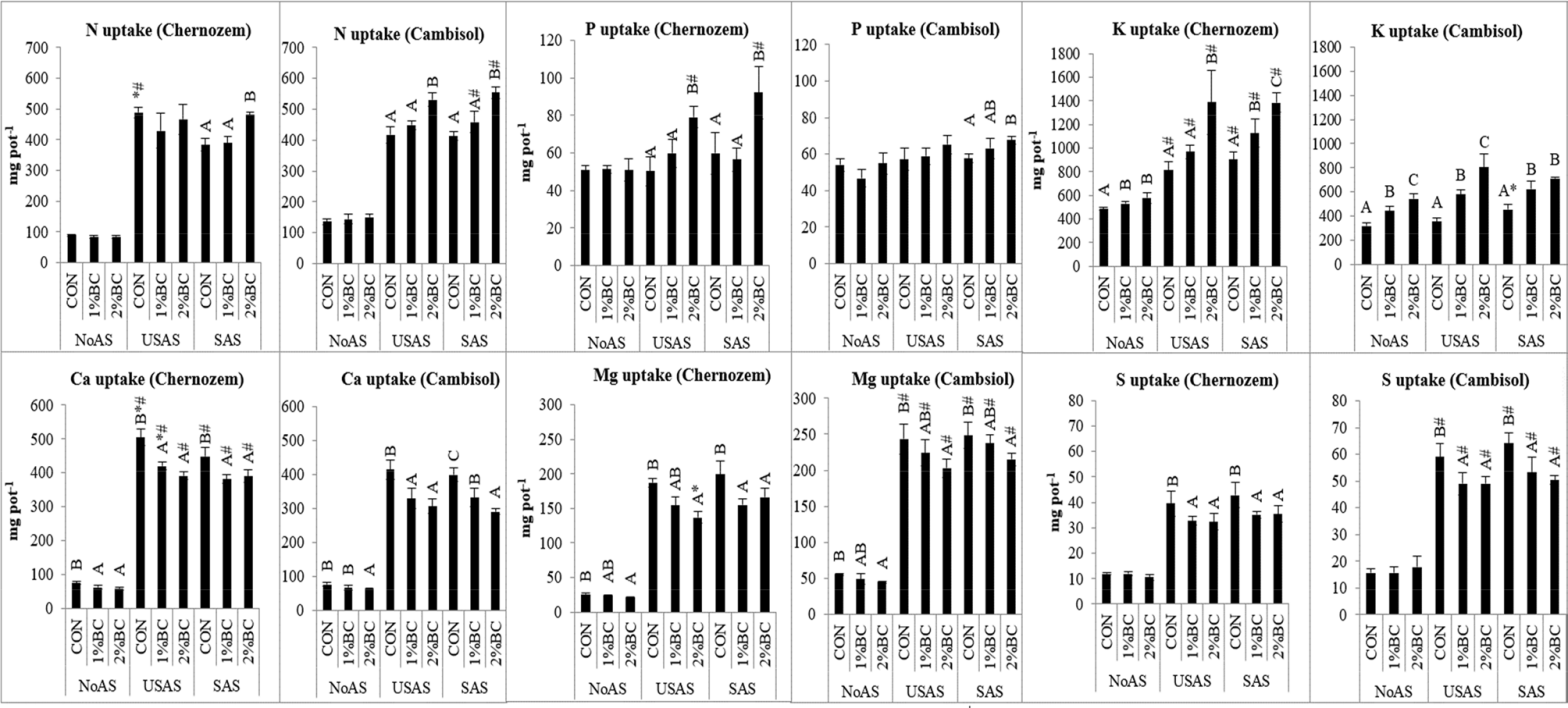
The uptake nutrients (N, P, K, Ca, Mg and S) by aboveground biomass of maize as affected by biochar, USAS (un-stabilized ammonium sulphate) and SAS (stabilized ammonium sulphate with DMPP). Different upper-case letters indicate a significant difference between variants within the same treatments of the same soil. *Represents a significant difference along biochar treatments of SAS and USAS (pair wised t-test between 1% BC of USAS and 1% BC of SAS and between 2% BC of USAS and 2% BC of SAS). ^#^Represents significant difference of pair wise t-test along different soils of similar treatments (CON of USAS Chernozem with CON of USAS Cambisol and likewise). *NoAS* No ammonium sulphate, *CON* control, *1% BC* 1% biochar, *2%BC* 2% biochar.

The application of biochar in NoAS and USAS treatments of the Chernozem was not able to induce any significant effect, while in the SAS treatments, the application of 2% biochar induced a significant (*p* = 0.05) increment of N uptake of 26% (Fig. 2). On the acidic Cambisol, the application of 2% BC induced a significant increment of N uptake in both the USAS and SAS treatments by 27 and 34%, respectively. In the Chernozem, the uptake of N in control treatment of USAS was higher than the control treatment of SAS. In the Cambisol, the uptake of N was significantly higher at the 1 and 2% BC treatments of SAS than the corresponding treatment of the Chernozem soil. The multivariate analysis of variance of the between-subject effects (SI S1) revealed that the highest effect was fertiliser (F = 1592, *p* < 0.001), then soil (F = 48.4, *p* < 0.001) and biochar (F = 40.7, *p* < 0.001) on the uptake of nitrogen. More interestingly, there was also a significant interaction effect for fertiliser and biochar (F = 13.4, *p* < 0.001), soil, biochar and fertiliser (F = 3.40, *p* < 0.015) and an interaction between soil and biochar (F = 12.1, *p* < 0.001) (SI S1). The application of 2% BC increased the NUE by 9.5% in the Chernozem with the SAS treatment and by 11 and 13.7% for the USAS and SAS treatments of the acidic Cambisol, respectively (Table 3).

**Table 3:** The effect of biochar on the use efficiency of nitrogen (NUE) and sulphur (SUE).

		NUE (%)		SUE (%)	
		Chernozem	Cambisol	Chernozem	Cambisol
USAS	1% biochar	− 5.84	3.11	− 0.59	− 0.88
	2% biochar	− 2.23	10.9	− 0.63	− 0.87
SAS	1% biochar	0.59	4.48	− 0.69	− 0.97
	2% biochar	9.47	13.7	− 0.66	− 1.22

Nutrient use efficiency in percentage calculated per added amount of N and S from fertilizer.

#### Phosphorus

In both soils, the application of biochar without fertiliser was not able to induce any significant changes in P uptake (Fig. 2). However, the application of 2% biochar with both USAS and SAS was able to induce significant (*p* < 0.05) increments of P uptake by 58 and 54%, respectively on the neutral Chernozem and significant (*p* < 0.05) increments of 14 and 18% in the USAS and SAS treatments of the acidic Cambisol, respectively. The uptake of P for the 2% BC treatments of USAS and SAS was significantly higher for the Chernozem soil than for the corresponding treatments of Cambisol. The application of fertiliser had the highest effect (F = 32.8, *p* < 0.001) on the uptake of P (SI S1). It was followed by the effect of biochar (F = 31.8, *p* < 0.001) and the interaction effect of soil with biochar (F = 8.29, *p* < 0.001), then the fertiliser with biochar (F = 6.81, *p* < 0.001). The interaction between soil, fertiliser and biochar also had a significant (F = 5.27, *p* < 0.001) effect.

#### Potassium

Soil type induced the highest effect (F = 333, *p* < 0.001) on the uptake of K, then fertiliser (F = 140, *p* < 0.001), biochar (F = 96.8, *p* < 0.001), the interaction of soil with fertiliser (F = 46.1, *p* < 0.001), fertiliser with biochar (F = 8.98, *p* < 0.001) and the interaction effect of soil, fertiliser and biochar (F = 2.55, *p* < 0.001) (SI S1). The application of biochar induced an increment of K uptake in all treatments of both soils. In particular, the application of 2% biochar induced a significant (*p* < 0.05) increment of K uptake in all treatments (NoAS, SAS and USAS) of both soils (Fig. 2), while a 1% application induced a significant increment in all treatments of Cambisol and SAS and USAS treatment of the Chernozem. The increment was higher in the case of the acidic Cambisol as compared to the neutral Chernozem. The uptake of K in the Chernozem soil CON, as well as the 1 and 2% BC of both USAS and SAS was significantly higher than corresponding treatments of the Cambisol soil (Fig. 2). The increment of K uptake in Chernozem was by 19, 70 and 53%. Meanwhile in the Cambisol, it was by 71, 127 and 57% at the 2% BC application rate in the NoAS, USAS and SAS, respectively.

#### Calcium

The highest impact on the uptake of Ca was obtained from the application of fertiliser (F = 2588, *p* < 0.001) then soil type (F = 148, *p* < 0.001), biochar (F = 104, *p* < 0.001), the interaction of soil with fertiliser (F = 44.9, *p* < 0.001) and fertiliser with biochar (F = 17.4, *p* < 0.001) (SI S1). The application of biochar decreased the uptake of Ca in all treatments (NoAS, SAS and USAS) of both soils (Fig. 2). In the case of the Chernozem, the application of both 1 and 2% biochar significantly (*p* < 0.05) reduced the uptake of Ca in all treatments (NoAS, SAS and USAS) (Fig. 2). The declines with the 2% of biochar rate were by 22, 22 and 13% for the NoAS, USAS and SAS, treatments, respectively. In the case of the acidic Cambisol, the decline was significant (*p* < 0.05) at both the 1 and 2% application of biochar for the SAS and USAS, while it was significant only at the 2% biochar application rate at NoAS treatments. The declines for the 2% BC rate were 17, 26 and 27% for the NoAS, USAS and SAS, respectively. The uptakes of Ca in the Chernozem soil CON as well as the 1 and 2% BC of both the USAS and SAS were significantly higher than corresponding treatments of the Cambisol soil (Fig. 2).

#### Magnesium

The highest effect on the uptake of Mg was obtained from the application of fertiliser (F = 1524, *p* < 0.001), then soil (F = 332, *p* < 0.001) and biochar (F = 38.2, *p* < 0.001), the interaction of soil with fertiliser (F = 19.2, *p* < 0.001) and fertiliser with biochar (F = 6.18, *p* < 0.001) (SI S1). As that of Ca, the uptake of Mg declined with the application of biochar in all treatments (NoAS, SAS and USAS) of both soils (Fig. 2). The application of 2% biochar induced a significant (*p* = 0.05) decline in all treatments of both soils, while only 1% caused a decline in the SAS and USAS of Chernozem soil. The declines in Chernozem soil at the 2% biochar rate were 17, 27 and 17% for NoAS, USAS and SAS, respectively; while in the Cambisol, the declines were 19, 17 and 13 for the NoAS, USAS and SAS, respectively. Inversely, the uptakes of Mg in the Cambisol soil CON as well as 1 and 2% BC for both USAS and SAS were significantly higher than the corresponding treatments for Chernozem soil (Fig. 2).

#### Sulphur

The highest effect on the uptake of S was from fertiliser (F = 708, *p* < 0.001) then soil (F = 296, *p* < 0.001) and the interaction of soil with biochar (F = 30.2, *p* < 0.001), biochar (F = 26.7, *p* < 0.001) and the interaction of fertiliser with biochar (F = 7.44, *p* < 0.001) (SI S1). The application of biochar without fertiliser was not able to induce any significant change in either soil, while both 1 and 2% biochar induced a significant decline in the SAS and USAS treatments of both soils (Fig. 2). The declines of S uptake in the Chernozem soil at 2% biochar application were by 18 and 17% in USAS and SAS, respectively and by 17 and 22% for USAS and SAS in the Cambisol, respectively. The S use efficiency from the applied fertiliser declined with the application of biochar in both the SAS and USAS treatments (Table 3). The decline was up to 0.63, 0.66 with 2% biochar in the USAS and SAS treatments of the Chernozem and up to 0.87, 1.22% in the USAS and SAS treatments of Cambisol, respectively. All USAS and SAS treatments in Chernozem soil had significantly higher uptakes of S than their respective treatments in Cambisol.

### pH and composition of soil solution

#### pH

**Figure 3:**
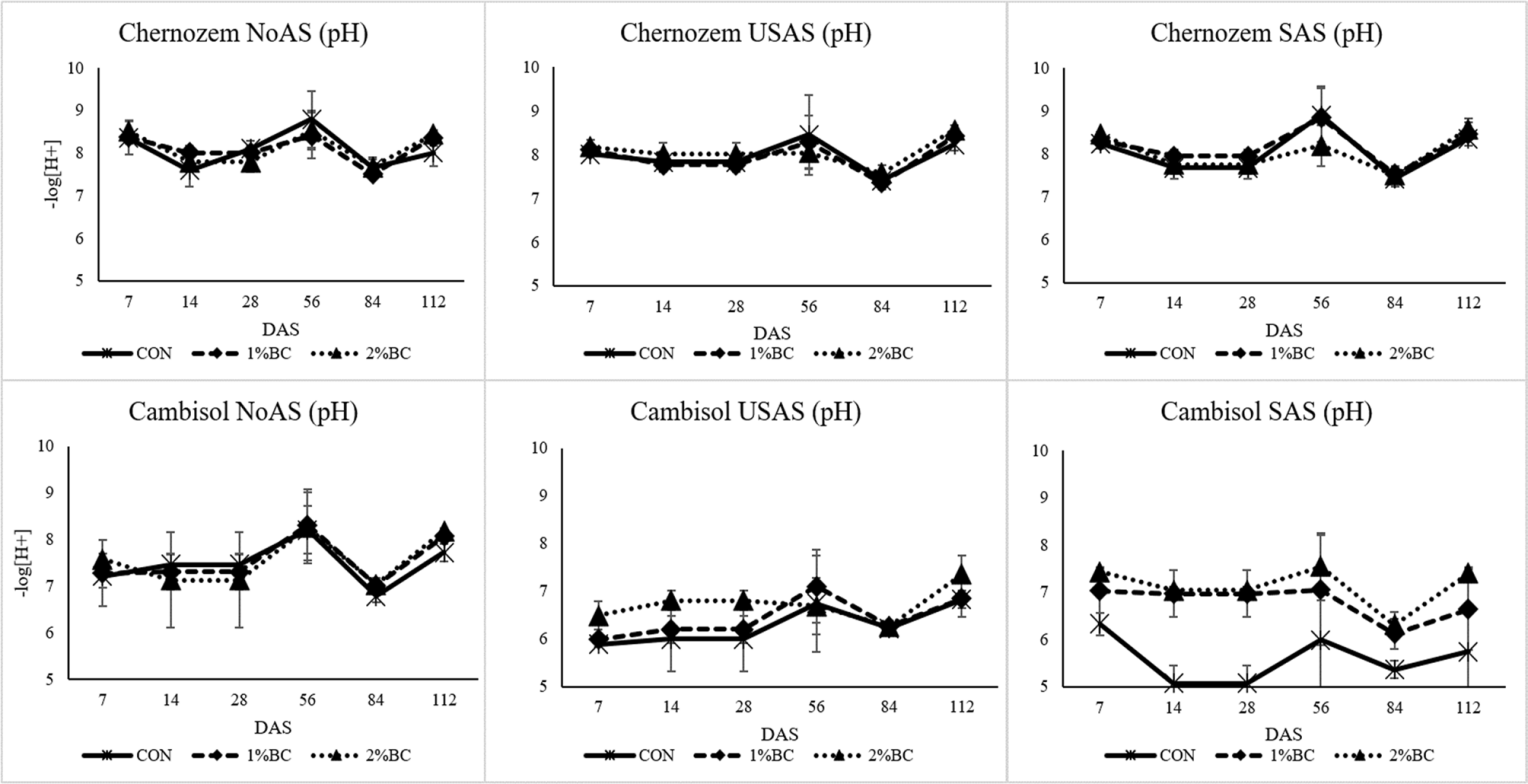
The effect of biochar, USAS (un-stabilized ammonium sulphate) and SAS (stabilized ammonium sulphate with DMPP) on the pH of soil solution, *NoAS* No ammonium sulphate, *CON* control, *1%BC* 1% biochar, *2%BC* 2% biochar, *DAS* day after sowing.

All the investigated factors soil, biochar, fertiliser, sampling period, the interaction of soil with biochar and the interaction of biochar with fertiliser had a significant effect on the pH of the soil solution. Based on the rANOVA, the source of highest variation was soil type with (F = 201, *p* < 0.001), then time of sampling (F = 20.2, *p* < 0.001) (SI S2). The effect of biochar on the pH of the soil solution was minimal in the case of the Chernozem soil, while in the case of the acidic Cambisol, co-application of biochar with AS significantly increased the pH of the soil solution at least by the seventh DAS (Fig. 3). At the seventh DAS, the highest increment in pH was in the SAS treatments of the acidic Cambisol, which was up to 1.11 units with the 2% biochar application.

#### Nitrate and ammonium

**Figure 4:**
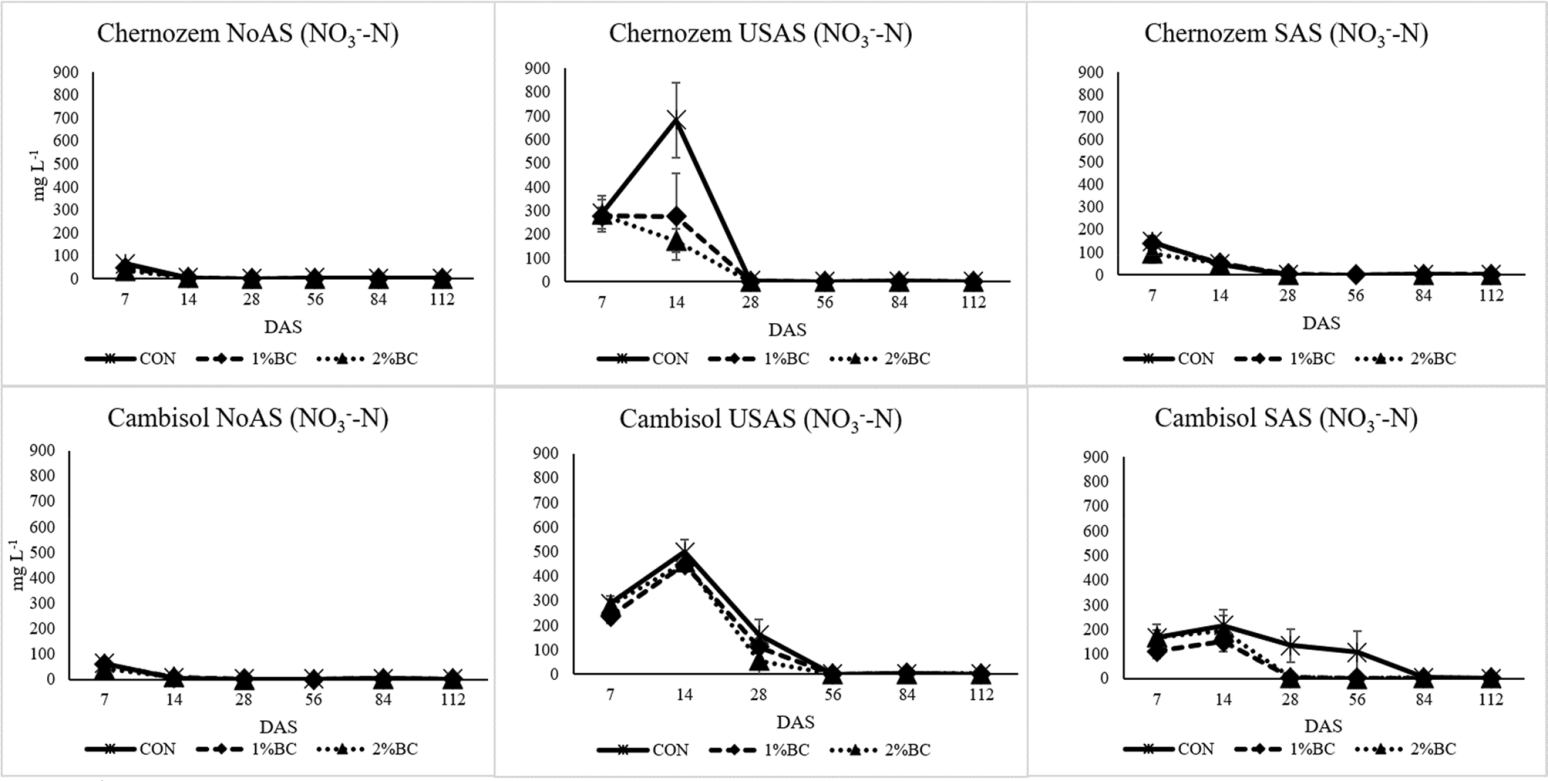
The effect of biochar, USAS (un-stabilized ammonium sulphate) and SAS (stabilized ammonium sulphate with DMPP) on the concentration of NO_3_^−^–N (mg L^−1^) in soil solution. *NoAS* No ammonium sulphate, *CON* control, *1%BC* 1% biochar, *2%BC* 2% biochar, *DAS* day after sowing.

**Figure 5:**
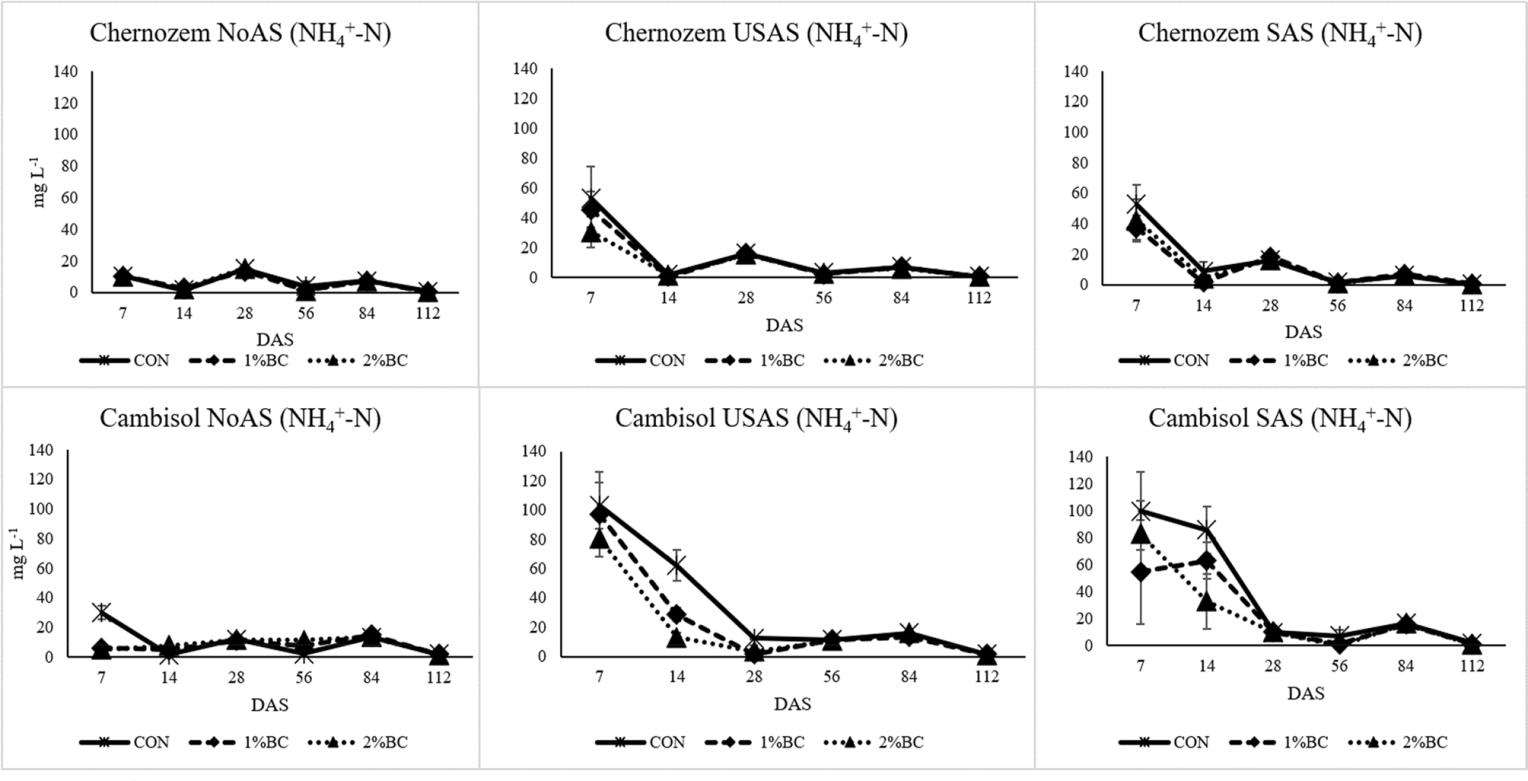
The effect biochar, USAS (un-stabilized ammonium sulphate) and SAS (stabilized ammonium sulphate with DMPP) on the concentration of NH_4_^+^–N (mg L^−1^) in soil solution. *NoAS* No ammonium sulphate, *CON* control, *1%BC* 1% biochar, *2%BC* 2% biochar, *DAS* day after sowing.

An increment of NO_3_^–^ N concentration was detected in the control treatment of USAS in the Chernozem and all USAS treatments of the Cambisol between 7 and 14 DAS, while in the remaining treatments, the concentration of NO_3_^–^ N was rather decreasing over time (Fig. 4). The rANOVA for the effect of factors on the concentration of NO_3_^−^–N in the soil solution is presented in SI S2. Based on the rANOVA (SI S2) soil type, biochar, fertiliser, sampling period and the interaction of biochar with fertiliser had a significant effect on the concentration NO_3_^−^–N in soil solution. The highest effect was attributed to the sampling period (F = 230, *p* < 0.00). The concentration of NH_4_^+^–N in soil solution was significantly affected by soil, biochar, fertiliser, the period of sampling and the interaction of soil with biochar (SI S2). At the seventh DAS, higher concentrations of NH_4_^+^–N were detected in the soil solution of SAS compared to USAS. Lower concentrations of NH_4_^+^–N in soil solution were found in variants treated by biochar application by at least the seventh DAS (Fig. 5). After harvesting the maize, the soils were analysed for the available fractions of mineral N (SI S3). The effect of biochar on both NO_3_^–^ N and NH_4_^+^–N was very negligible in the Chernozem soil except a significant decline in the NO_3_^–^ N content of the NoAS treatment. In the case of the Cambisol, the application of 2% biochar significantly decreased the content of soil NH_4_^+^ and increased the content of NO_3_^−^ in the USAS and SAS treatments.

#### Phosphorus and sulphur

**Figure 6:**
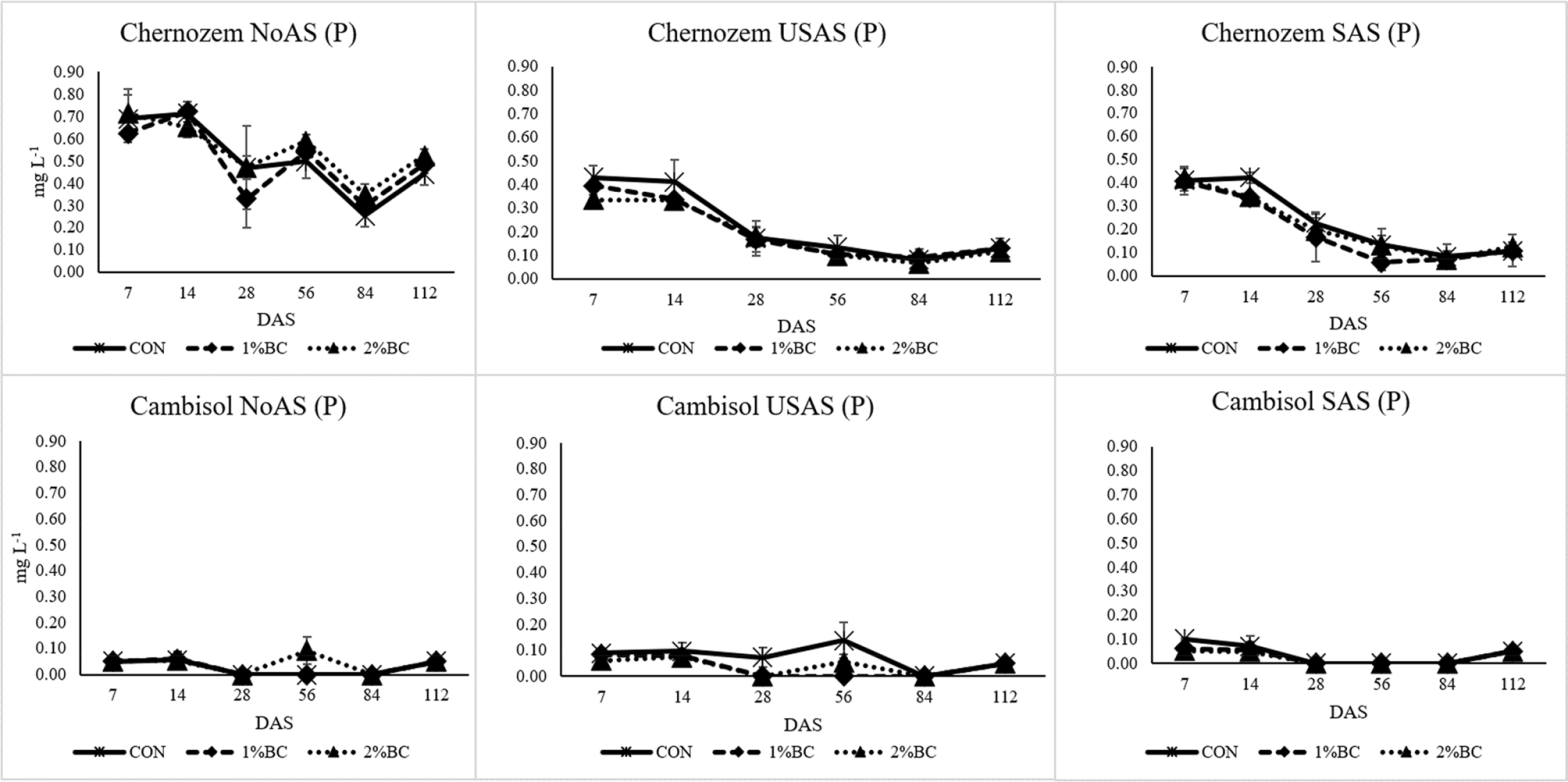
The effect of biochar, USAS (un-stabilized ammonium sulphate) and SAS (stabilized ammonium sulphate with DMPP) on the concentration of P (mg L^−1^) in soil solution. *NoAS* No ammonium sulphate, *CON* control, *1%BC* 1% biochar, *2%BC* 2% biochar, *DAS* day after sowing.

The concentration of P was significantly higher in the Chernozem soil as compared to the Cambisol (Fig. 6). Only the main factors soil, fertiliser and time of sampling induced a significant effect on the concentration of soil solution P (SI S2). The highest effect was attributed from time of sampling with F = 193, *p* < 0.001. In all treatments, the concentration of P had a decreasing trend over time and a slightly lower concentration was detected in biochar-treated soils, especially at seven DAS. The application of AS fertiliser increased the concentration of S in soil solution. The effect of biochar on the concentration of S was not very noticeable except for a slight decline in SAS and USAS treatments by the seventh DAS (SI S4). All the investigated factors except the interaction of soil with biochar had a significant effect on the concentration S in soil solution (SI S2). The greatest effect was from the application of fertiliser (F = 250, *p* < 0.001).

#### Potassium, calcium and magnesium

All the factors investigated, except the interaction of biochar with fertiliser, had a significant effect on the concentration of K in soil solution, with the highest effect arising from the period of sampling F = 744, *p* < 0.001 (SI S2). A higher concentration of K was in the Cambisol than in the Chernozem soil. Application of AS fertilisers increased the concentration of K in soil solution, especially at DAS 7, and decreased over time. Furthermore, the application of BC resulted in higher K concentrations in soil solution, but this effect was detectable only at the early stage of the experiment (SI S5). Similarly, the application of AS fertiliser significantly increased the concentration of Ca and Mg in the soil solution of both soils, by at least the 7th day of sampling. Among the investigated factors, biochar, fertiliser, time of sampling and the interaction of biochar with fertiliser had a significant effect on the concentration of Ca (SI S2). The highest effect was from the time of sampling with F = 442, *p* < 0.001. The concentration of Ca showed a decline over time except for a significant increment in control treatment of USAS Ca from 959 mg L^−1^ (7 DAS) to 1555 mg L^−1^ (14 DAS) in the Chernozem (SI S6). In the case of Mg, only the main factors soil, fertiliser and time of sampling induced a significant effect on the concentration of soil solution Mg (SI S2). The highest effect was from the application of fertiliser with F = 267, *p* < 0.001. Again, the concentration of Mg showed a decline over time except for a significant increment in the control treatment of USAS from 43 mg L^−1^ (7 DAS) to 65 mg L^−1^ (14 DAS) (SI S7).

## Discussion

### Mechanisms of NUE improvement

The highest maize aboveground biomass was achieved by the combination of 2% BC and stabilised ammonium sulphate (SAS) identically on both soils (Fig. 1). Moreover, the application of BC was effective to increase maize biomass only in combination with SAS. Based on the between-subject effects analysis of variance (SI S1), the factor with the highest influence on the uptake of N and maize biomass was the fertiliser, which is due to the N supplied. The significant increment of maize biomass at the co-application of biochar and DMPP treated ammonium sulphate was mainly due to the increment of N uptake and improved NUE as the highest increments of N uptake (34%) and NUE (13.7%) were in the treatment of SAS combined with 2% BC. The increment of maize biomass, improved uptake of N and NUE when the biochar was co-applied with the SAS indicates the positive association effect of biochar with DMPP. One of the mechanistic reasons for the improved yield and NUE in these treatments could be due to further delay of the nitrification by biochar. Therefore, biochar could further extend, or delay the nitrification inhibition induced by DMPP and slows the release of nitrate for the later stage of maize growth^37^. This output is in complete disagreement with the findings of Sheikhi et al.^38^, Fuertes-Mendizábal et al.^39^, and Keiblinger et al.^40^, where authors presented the negative interaction of DMPP with biochar.

Other important finding is that the biochar-induced increment of NUE was higher in the acidic Cambisol fertilized by DMPP treated ammonium sulphate. This could indicate the better interaction of biochar with DMPP treated ammonium sulphate in the acidic soils as compared to neutral or alkaline soils. The first reason for the better joint effect of biochar with DMPP treated ammonium sulphate in the acidic Cambisol could be due to the acidic pH (4.8) and higher sand content of the Cambisol (26.1%) than the Chernozem (13.2%). The short delay of NH_4_^+^ oxidation by DMPP in soil with a higher proportion of clay is expected due to the sorption of DMPP by clay minerals and their reduced effect^41^, while the opposite is true in soils with a high proportion of sand and further prolongation by biochar is expected. Secondly, the nitrification inhibitory effect of DMPP is much higher in acidic soil as compared to alkaline soils^42^, which is again further prolonged by the application of biochar. This was noticeable for the USAS and SAS treatments of Cambisol at 7 and 14 DAS (Fig. 5). This all leads to a low rate of NH_4_^+^ oxidation to NO_3_^−^ and the subsequent slow release for the later stage of maize growth. Moreover, even if we expect some excess nitrification in this soil, the nitrate loss due to leaching could be very low as adsorption of nitrate by biochar at the acidic pH of the Cambisol is higher than the neutral pH of the Chernozem soil. This is because of the more favoured adsorption of NO_3_^−^–N in the acidic soil conditions^24^. This statement agreed with the NO_3_^−^–N and NH_4_^+^–N contents in the 0.01 M CaCl_2_ extraction of soil samples collected after the harvest of maize (SI S3). There was a significantly higher concentration of NO_3_^−^–N in the Cambisol with 2% biochar with SAS and USAS treatments after the harvest of maize than in the controls (no biochar), whereas there was a significant decline in the content of NH_4_^+^–N. This was not true in the case of the neutral Chernozem soil, which indicates that NH_4_^+^ was being slowly nitrified in the biochar treatments of acidic Cambisol, especially in the SAS and USAS treatments accompanied by NO_3_^–^–N availability even after the harvest of the maize, which is beneficial for the next cropping season.

### The uptake and use efficiency of sulphur

The uptake of S was higher in the SAS and USAS treatments of the Cambisol than that of the Chernozem. This is in agreement with the high content of available S in the Cambisol soil solution of the USAS and SAS treatments. The application of biochar induced a decline in the uptake of S in the SAS and USAS treatments. The application of biochar in the fertilised treatments reduced sulphur use efficiency up to 1.22%, meaning that there were always lower uptakes of S in the biochar treatments of SAS and USAS than in the controls in both soils. This is mainly due to the low availability of S in the soil solution of biochar treatments (SI S4). The decline in the availability of S from biochar in treatments of SAS and USAS could be due to the precipitation of Ca released from biochar with sulphate and the adsorption by biochar. This is because the same trend was shown for the Ca uptake, which declined with the biochar application and nevertheless gave a higher Ca depletion in the soil solution of biochar treatment of USAS and SAS. The decline of Ca and S in soil solution was significantly (*p* = 0.05) correlated (r = 0.95) in the Chernozem soil and (r = 0.89) in Cambisol. This phenomenon could indicate that the co-precipitation of Ca and S resulted in the decline of both Ca and S in the biochar treatments of USAS and SAS. The increment in S adsorption and the formation of S-Ca precipitate in the Ca-rich condition is evident^43^. Biochar could also decrease the availability of S due to the sorption of SO_4_^2−^ by electrostatic interaction with the charged surface of biochar^44^. The decline in the content of sulphate by biochar application has been reported due to the formation of weakly soluble CaSO_4_^45^.

The content of S in the treatment without fertiliser was lower compared to ammonium sulphate treatments (SAS and USAS). This is mainly due to the release of sulphate from applied ammonium sulphate and the improved uptake of S in the soils having higher contents of N. The decrease of sulphur uptake in low N available conditions has been described by Clarkson et al.^46^. Again, the multivariate analysis (SI S1) confirmed a higher effect of fertiliser (F = 708, *p* < 0.001) compared to soil type. There was also a significant interaction effect of biochar and fertiliser (F = 7.44, *p* < 0.001) revealing the highest effect of biochar to reduce the uptake of S is in fertilised treatments, while it had insignificant effects in NoAS treatments.

### The uptake of phosphorus

The uptake of P was generally higher in the Chernozem soil than in the Cambisol due to the higher availability and total content of P in Chernozem soil (Table 1) and a subsequent significant higher concentration of P in the soil solution (Fig. 6). The single application of biochar without N fertiliser was not able to induce significant changes in P uptake. However, biochar was able to increase the P uptake in the USAS and SAS treatments of both soils without a detectable increment of P in soil solution. This is likely not due to the release of P from biochar, as the applied biochar does not have available P for plant uptake (Table 1). In similar studies, the application of biochar at the rate of 10 t ha^−1^ was able to increase maize P uptake^47^. The main reason for the increment of P uptake could be the biochar-induced weakening/inhibition of phosphate anions (H_2_PO_4_^−^, HPO_4_^2−^ or PO_4_^3−^) adsorption by the Al/Fe (hydr)oxides of soils^48^. The adsorption of soil HPO_4_^2−^ or PO_4_^3−^ by Fe (hydr)oxides is expected to be lower at the relatively higher pH induced by biochar. This is due to the repulsion of negatively charged HPO_4_^2−^ and/or PO_4_^3−^ by the negatively charged surface sites of the ferrihydrite and as a result of OH^−^ ion competition on the negatively charged sorption sites at the higher soil pH induced by biochar^49^. The increment of soil pH due to the release of Ca^2+^, Mg^2+^ and K^+^ from biochar could effectively reduce the solubility of reactive Al^3+^ oxides, and this could reduce the sorption of P in acidic soil^50^. The biochar-induced increment of soil pH is evident and significant especially in the SAS and USAS treatments of acidic Cambisol at least at the seventh DAS (Fig. 3). Biochar could increase soil pH by releasing exchangeable base cations (Ca^2+^, K^+^ and Mg^2+^) and their subsequent replacement by the exchangeable Al^3+^ and H^+^ on the exchangeable sites of biochar as well as the binding of surplus H^+^ ion to the negatively charged (carboxylic, hydroxyl and phenolic) surface functional groups of biochar^15,51^. Thus, the better expression of pH increment in the SAS and USAS treatments of acidic Cambisol than the neutral Chernozem is simply due to the greater effectiveness of biochar to increase soil pH in soils having low pH, CEC and exchangeable Ca^2+^.

### The uptake of potassium, calcium and magnesium

Based on the multivariate analysis of variance (SI S1), the highest factor affecting the uptake of K was soil due to the higher uptake of K in the Chernozem soil with the higher content of both total and available K content than the Cambisol (Table 1) and their release to the soil solution. The second highest factor influencing the uptake was the application of ammonium sulphate. The reason for the higher uptake of K in the ammonium sulphate treatments is the higher availability of K induced by the displacement of exchangeable K^+^ to the soil solution by NH_4_^+^ from the applied ammonium sulphate (SI S3). Based on the study of Wang et al.^52^, the application of AS increased the content of water-soluble K up to 160%, while the exchangeable content of K^+^ declined by up to 19%, supporting the release of K^+^ into the soil solution due to the displacement from the exchangeable site of soils by NH_4_^+^. The third significant effect was from the application of biochar. The application of 2% biochar was able to induce a significant increment of K uptake in all treatments of both soils. The improvement of K uptake in the biochar-amended treatments of our soil is expected due to much higher CaCl_2_ (0.01 M) and extractable contents of K (2278 mg kg^−1^) from the biochar used in this study compared to the Chernozem (65 mg kg^−1^) and Cambisol (32 mg kg^−1^) soils. Biochar could serve as a potential source of K, and this results in the subsequent increment of K uptake^53^. The release and improvement of K uptake by crops after biochar application have been previously reported^54,55^. Similarly, the improvement of K availability and the subsequent increment of K uptake by maize was reported after the application of 2% vineyard pruning biochar^56^.

The effect of biochar on the uptake of Ca and Mg was opposite to the uptake of K. The application of biochar decreased the uptake of both Ca and Mg. The decline in the uptake of both elements with biochar application is due to the antagonistic effect of K uptake. This agrees with all treatments; declines in the Ca and Mg concentrations were noticeable in all treatments where there was an increment of K uptake. The increment of K availability by biochar application could induce a reduction of Ca and Mg uptake due to the blockage of non-specific Ca and Mg transporters by the uptake of K. Therefore, the competition of K for transporters and preferential uptake of K in the K rich soil solution induces a reduction of Ca and Mg uptake^57^. The study of Horie et al.^58^ confirmed that the class II high-affinity potassium transporter (HKT) was involved in the transport of K, Ca and Mg, and hence preferentially transporting K over the divalent cations (Ca^2+^ and Mg^2+^), leading to the suppression of Mg and Ca uptake in K-rich environment. The highest effect of fertiliser (SI S1) on the uptake of Ca and Mg is linked to the higher maize biomass in the fertilised treatments, which was 5 times higher than the control and increment of Ca and Mg in soil solution (SI S6 and S7). The increment of available Ca and Mg content in fertilised treatment of soil solution is again caused by the displacement of exchangeable Ca^2+^ and Mg^2+^ from the exchangeable site of soils by NH_4_^+^^59^. The effect of NH_4_^+^ on the displacement of Ca^2+^ and Mg^2+^ from the exchangeable site of soil can be observed from the increased concentration of Ca and Mg in the USAS and SAS treatments compared to control in both soils (SI S6 and S7). Similarly, the oxidation of NH_4_^+^ to nitrate is known to release 2H^+^ ion^60^. Thus, the temporary increment of Ca and Mg in USAS and SAS treatments of both soil could be also associated with the replacement Ca^2+^ and Mg^2+^ by the H^+^ ion released from the nitrification result of (NH_4_)_2_SO_4_. Further biochar induced a decline of Ca content in the soil solution of the neutral Chernozem and an increment in the acidic Cambisol. Biochar is principally capable of increasing available Ca content in soils having lower original Ca content than the biochar used, while biochar could induce a decline of Ca content when added to soils having higher Ca contents than the biochar applied^15^. The neutral Chernozem had a much higher content of exchangeable Ca^2+^ (253 mmol kg^−1^) than the acidic Cambisol (72 mmol kg^−1^) and biochar (176 mmol kg^−1^) (Table 1). Thus, when this type of biochar was added to the neutral Chernozem, we would expect a decline of Ca content, while incrementing in the acidic Cambisol.

### Mechanisms of biochar interaction with ammonium sulphate treated by DMPP

As discussed above, the positive impact of high temperature produced biochar co-application with DMPP treated ammonium sulphate fertilizer on the NUE and biomass of maize is mainly attributed due to the weak adsorption of NH_4_^+^ by the high temperature produced biochar (700 °C) used in this study. Therefore, the weakly adsorbed NH_4_^+^ could slowly nitrify and become available for the plant uptake at the later crop growing stages. This directly goes with the intended use of DMPP, which slows the nitrification of NH_4_^+^. Some previously published works of other studies seem quite opposing to our finding in some ways, which is attributed only to the higher production temperature of biochar (700 °C) used in this study. For example, the adsorption of DMPP by lower temperature produced biochar was reported for the biochars pyrolyzed at 450 °C^38^, 500 °C^39^, 400 °C and 525 °C^40^. In those studies, the presence of low NH_4_^+^ concentration in the treatments containing DMPP with biochar seems holding back the intended use of DMPP to limit the process of nitrification. However, the low availability of NH_4_^+^ in soils where low temperature produced biochar was applied is expected due to the strong sorption of NH_4_^+^ by easily available negatively charged oxygen containing functional groups of low temperature produced biochar^23,61^. Further, the lower temperature produced biochar can adsorb DMPP. Fuertes-Mendizábal et al.^39^ reported the adsorption of DMPP driven by the oxygen containing functional groups, more specifically carboxyl groups, of the lower temperature produced biochar (500 °C) used in their study. This is in the agreement with the finding of Keiblinger et al.^40^, reported a greater adsorption of DMPP by the biochar produced at 400 °C than the biochar produced from the same feedstock at the higher temperature (525 °C). The occurrence of the phenomena (adsorption of NH_4_^+^ and DMPP by biochar) is expected to be very low in our study due to the loss of oxygen containing functional groups proportional with the rise in production temperature. The clear decline of oxygen containing functional groups with the rise in temperature is evident^62^. Therefore, the use of high temperature produced biochar is a choice for the overall better performance of biochar-DMPP combination.

## Conclusion

The interaction effect of biochar with ammonium sulphate containing DMPP (NovaTec Solub 21) on the biomass and yield component of maize was studied on two soils with contrasting properties. The outcome revealed the effectiveness of biochar co-application with ammonium sulphate containing DMPP to induce a significant increment of maize biomass as well as the uptake of N, P and K.

Co-application of biochar with ammonium sulphate containing DMPP was able to increase maize biomass by 10%, nitrogen use efficiency by 13.7%, the uptake of P by 54%, and the uptake of K by 57% compared to a single application of ammonium sulphate containing DMPP. The interaction of biochar with ammonium sulphate containing DMPP was more effective to increase maize biomass, N uptake and K uptake in the acidic Cambisol, while P uptake increased in the neutral Chernozem. The application of biochar also induced a decline in the uptake of Ca and Mg because of the antagonistic effect of K. Additionally, biochar induced a decline of S uptake when co-applied with ammonium sulphate. In the case of un-stabilized ammonium sulphate, biochar was not able to induce a significant change in maize biomass, while there was an increment in N uptake only in the acidic Cambisol, an increment in the uptake of K in both soils and a decline in the uptake of Ca, Mg and S. Furthermore, the effect of biochar was also pronounced in the soil solution by increasing the concentrations of K, Mg in the soil solution of both soils, while there was an increment of Ca in the acidic Cambisol and a decline in the neutral Chernozem.

Generally, the interaction effect of biochar on the maize biomass, NUE and uptake of N was much higher when combined with ammonium sulphate containing DMPP than its co-application with un-stabilized ammonium sulphate and a single application of both stabilised and un-stabilized ammonium sulphate. Hereafter, we conclude that the application of high temperature produced biochar with ammonium sulphate containing DMPP could increase crop yield and improve nitrogen use efficiency due to a greater extent by the reduction of nitrogen losses.

## Supplementary Information

Supplementary Information
