# Supplementary material for: Co-application of high temperature biochar with 3,4-dimethylpyrazole-phosphate treated ammonium sulphate improves nitrogen use efficiency in maize
Source: Sci Rep. 2021 Mar 11;11:5711. doi: 10.1038/s41598-021-85308-0 (PMC7952707; doi:10.1038/s41598-021-85308-0)
Supplement: Supplementary file 1 — Supplementary Information [file 41598_2021_85308_MOESM1_ESM.pdf]

Supporting information (SI)

The interaction effect of biochar with stabilised and un-stabilised ammonium sulphate on the maize growth performance, nutrient use efficiency and chemistry of soil solution

Niguss Solomon Hailegnaw*[1] • Filip Mercl[1] • Martin Kulhánek[1] • Jiřina Száková[1] • Pavel Tlustoš[1]

[1]Department of Agro-Environmental Chemistry and Plant Nutrition, Faculty of Agrobiology, Food and Natural Resources, Czech University of Life Sciences Prague, Kamýcká 129, 16500 Prague 6 - Suchdol, Czech Republic

*Corresponding author

hailegnaw_niguss@af.czu.cz

SI 1. Multivariate analysis of variance for the biomass, total uptake of N, P, K, Ca, Mg and S with the single and interaction effect of factors (Soil, Biochar, and fertilizer).

		Soil	Fertilizer	Biochar	Soil * Fertilizer	Soil * Biochar	Fertilizer * Biochar	Soil * Fertilizer * Biochar
Biomass	F	131	5391	11.9	3.02	1.43	5.26	.489
	DF	1	2	2	2	2	4	4
	p	*.000*	*.000*	*.000*	*.057*	*.249*	*.001*	*.744*
N	F	48.4	1592	40.7	9.65	12.2	13.4	3.40
	DF	1	2	2	2	2	4	4
	p	*.000*	*.000*	*.000*	*.000*	*.000*	*.000*	*.015*
P	F	3.61	32.8	31.8	2.08	8.29	6.81	5.28
	DF	1	2	2	2	2	4	4
	p	*.063*	*.000*	*.000*	*.135*	*.001*	*.000*	*.001*
K	F	333	140	96.8	46.1	2.31	8.98	2.55
	DF	1	2	2	2	2	4	4
	p	*.000*	*.000*	*.000*	*.000*	*.109*	*.000*	*.050*
Ca	F	148	2588	104	44.9	1.30	17.4	2.07
	DF	1	2	2	2	2	4	4
	p	*.000*	*.000*	*.000*	*.000*	*.280*	*.000*	*.098*
Mg	F	332	1524	38.2	19.2	2.68	6.18	1.85
	DF	1	2	2	2	2	4	4
	p	*.000*	*.000*	*.000*	*.000*	*.078*	*.000*	*.133*
S	F	296	708	26.7	30.2	.68	7.44	1.22
	DF	1	2	2	2	2	4	4
	p	*.000*	*.000*	*.000*	*.000*	*.510*	*.000*	*.314*

The italicized values indicate the significance level.
DF; degree of freedom, F; F test and *p*; p value.

SI 2. Repeated Measure Analysis of variance rANOVA on the content of soil solution collected from the pots where the maize grown.

		Soil	Biochar	Fertilizer	Biochar * Soil	Biochar * Fertilizer	DAS
pH	F	201	5.97	14.6	4.51	4.19	20.2
	DF	1	2	2	2	4	5
	p	*0.00*	*0.00*	*0.00*	*0.01*	*0.00*	*0.00*
NO_3^- - N	F	25.5	12.5	81.7	0.14	3.66	230
	DF	1	2	2	2	4	5
	p	*0.00*	*0.00*	*0.00*	*0.87*	*0.01*	*0.00*
NH_4^+ - N	F	88.9	9.81	49.0	3.92	1.92	164
	DF	1	2	2	2	4	5
	p	*0.00*	*0.00*	*0.00*	*0.03*	*0.12*	*0.00*
P	F	165	0.21	27.1	0.18	0.22	193
	DF	1	2	2	2	4	5
	p	*0.00*	*0.81*	*0.00*	*0.84*	*0.92*	*0.00*
K	F	99.4	9.53	96.0	4.51	0.43	744
	DF	1	2	2	2	4	5
	p	*0.00*	*0.00*	*0.00*	*0.02*	*0.79*	*0.00*
Ca	F	3.41	17.6	357	1.24	5.80	442
	DF	1	2	2	2	4	5
	p	*0.07*	*0.00*	*0.00*	*0.30*	*0.00*	*0.00*
Mg	F	49.6	0.44	85.3	0.10	1.19	267
	DF	1	2	2	2	4	5
	p	*0.00*	*0.64*	*0.00*	*0.91*	*0.32*	*0.00*
S	F	19.5	8.01	250	1.13	3.57	169
	DF	1	2	2	2	4	5
	p	*0.00*	*0.00*	*0.00*	*0.33*	*0.01*	*0.00*

The italicized values indicate the significance level.
DF; degree of freedom, F; F test and *p*; p value

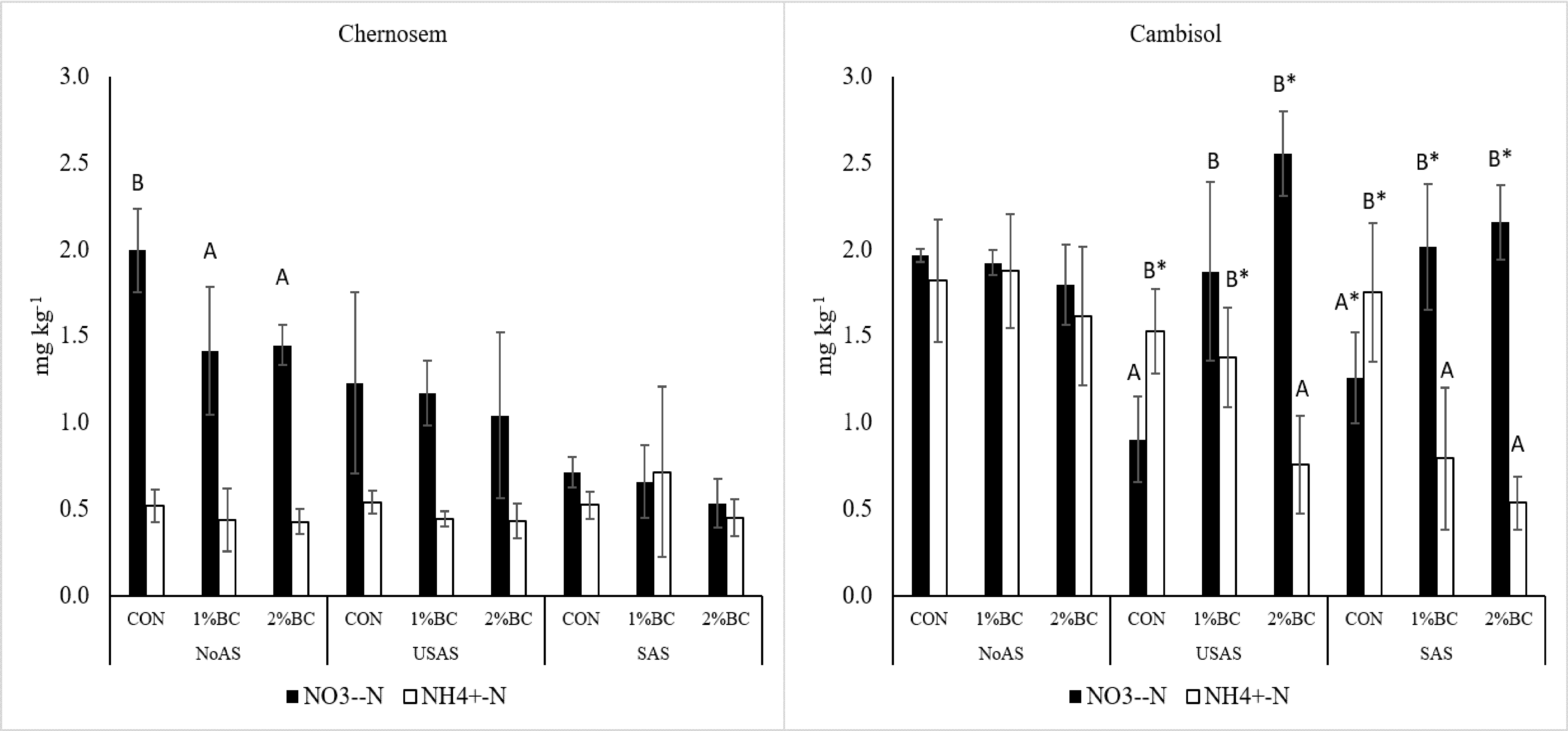

SI 3. The effect of biochar, USAS (un-stabilized ammonium sulfate) and SAS (stabilized ammonium sulfate) on soil content of NO_3^- - N and NH_4^+- N (mg L^{-1}). NoAS: No ammonium sulfate, CON: control, 1%BC: 1% biochar, 2%BC: 2% biochar. Different upper case letters indicate a significant difference between variants within the same treatments of the same soil. * represents significant difference of pair wise t-test along different soils of similar treatments (CON of USAS Chernozem with CON of USAS Cambisol and likewise).

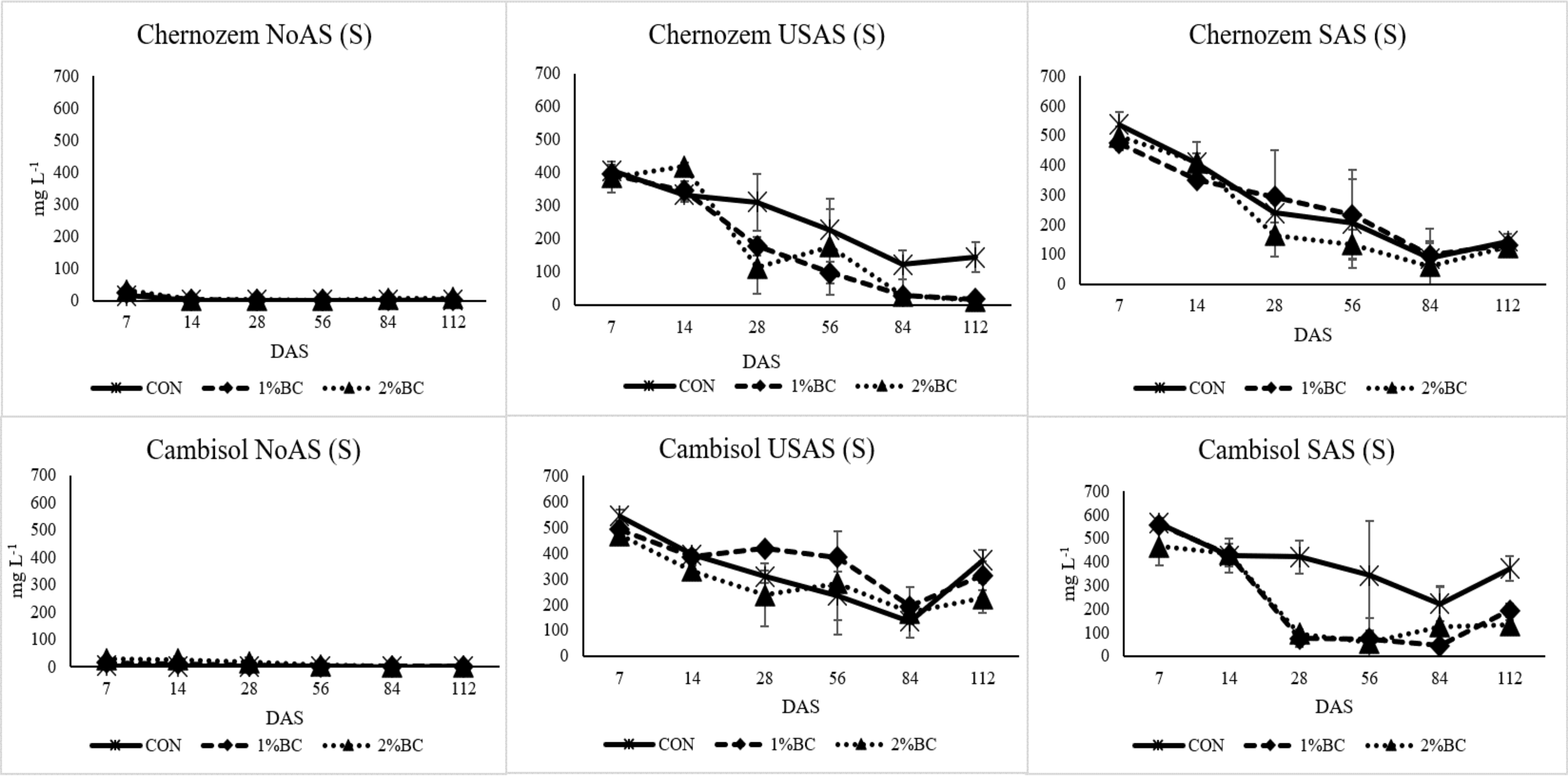

SI 4. The effect of biochar, USAS (un-stabilized ammonium sulfate) and SAS (stabilized ammonium sulfate with DMPP) on the concentration of S (mg L^{-1}) in soil solution. NoAS: No ammonium sulfate, CON: control, 1%BC: 1% biochar, 2%BC: 2% biochar, DAS: day after sowing.

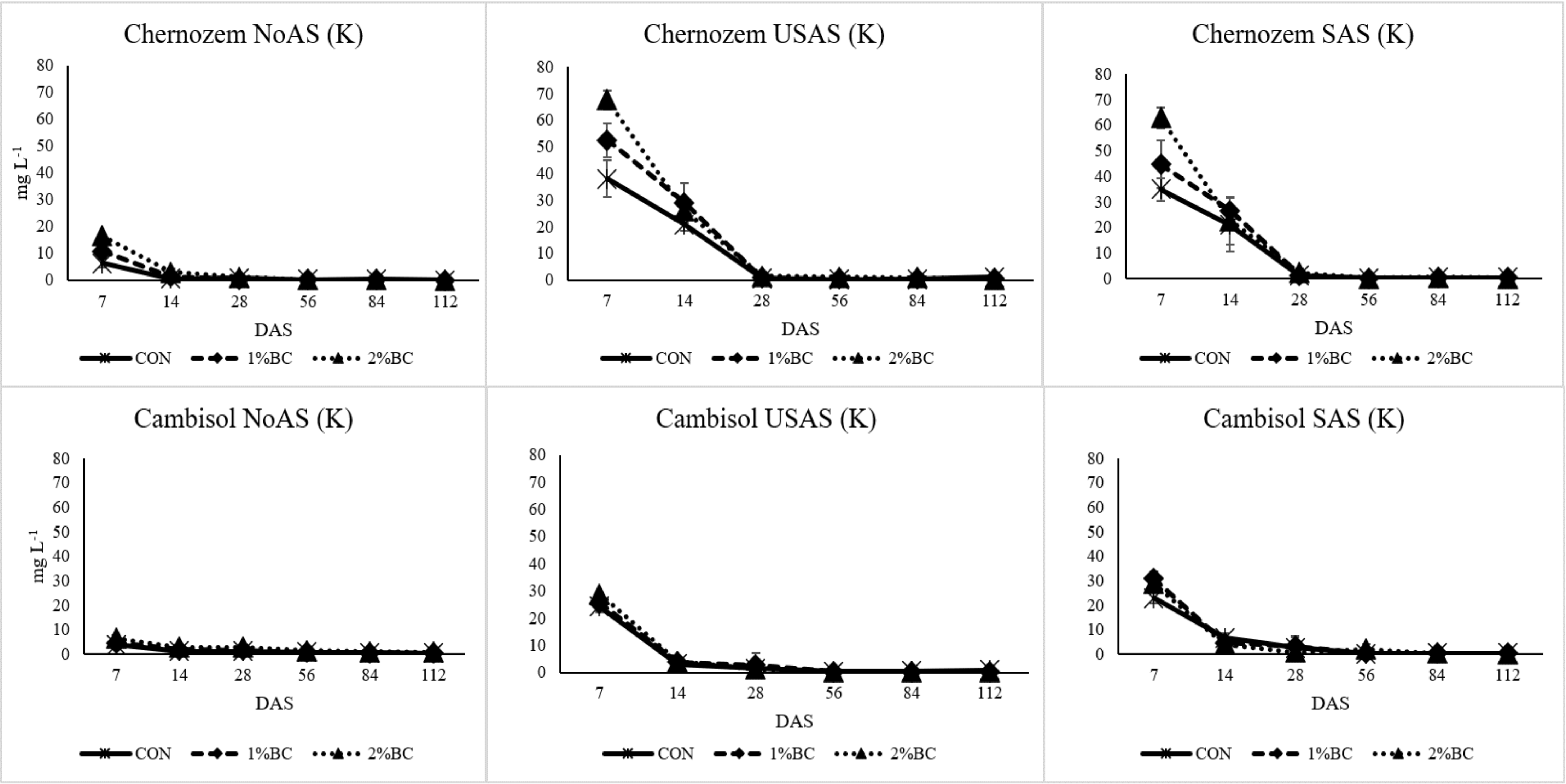

SI 5. The effect of biochar, USAS (un-stabilized ammonium sulfate) and SAS (stabilized ammonium sulfate with DMPP) on the concentration of K (mg L^{-1}) in soil solution. NoAS: No ammonium sulfate, CON: control, 1%BC: 1% biochar, 2%BC: 2% biochar, DAS: day after sowing.

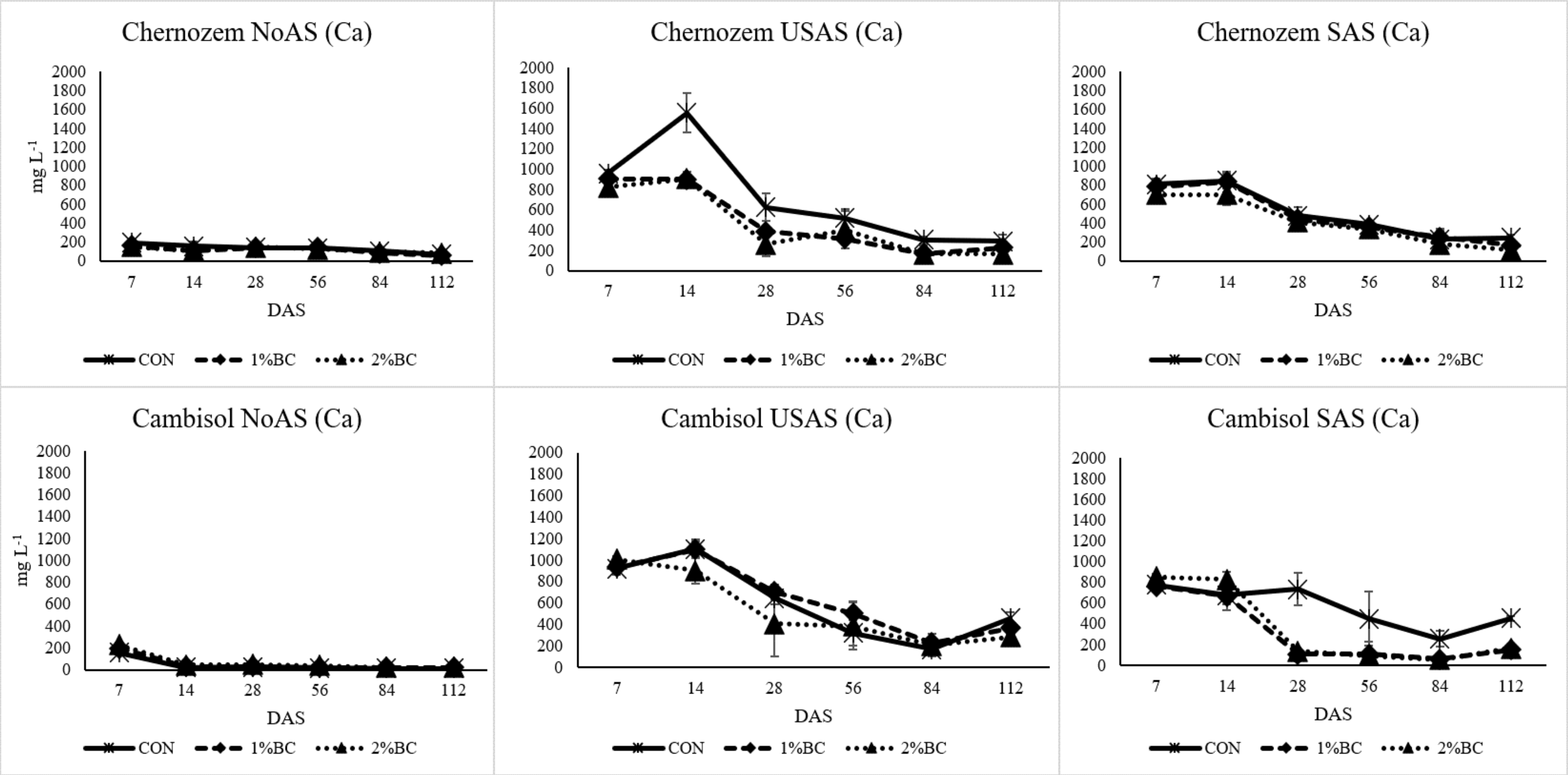

SI 6. The effect of biochar, USAS (un-stabilized ammonium sulfate) and SAS (stabilized ammonium sulfate with DMPP) on the concentration of Ca (mg L^{-1}) in soil solution. NoAS: No ammonium sulfate, CON: control, 1%BC: 1% biochar, 2%BC: 2% biochar, DAS: day after sowing.

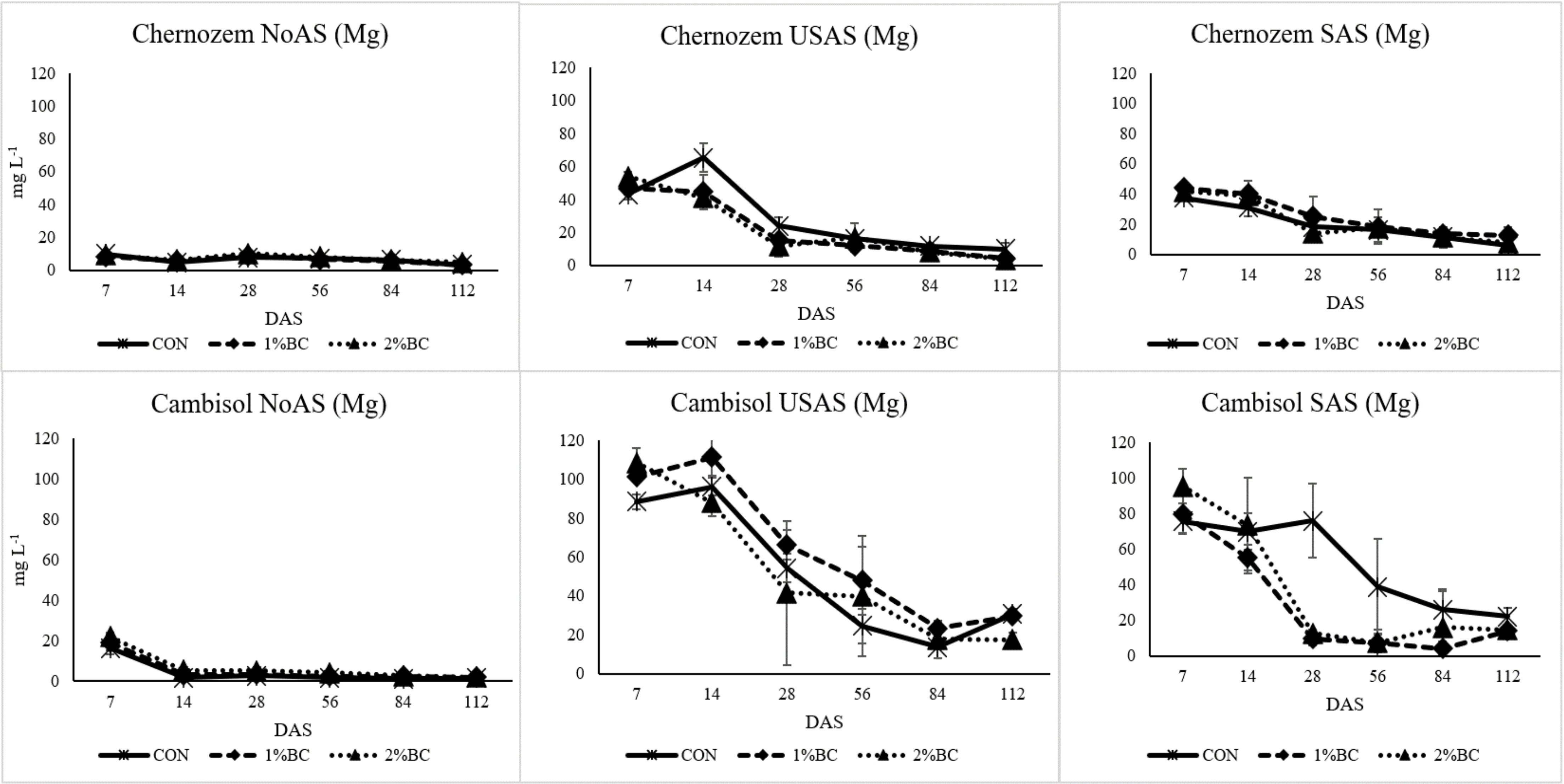

SI 7. The effect of biochar, USAS (un-stabilized ammonium sulfate) and SAS (stabilized ammonium sulfate with DMPP) on the concentration of Mg (mg L^{-1}) in soil solution. NoAS: No ammonium sulfate, CON: control, 1%BC: 1% biochar, 2%BC: 2% biochar, DAS: day after sowing.
